# Optimization of Sulfide/Sulfite Pretreatment of Lignocellulosic Biomass for Lactic Acid Production

**DOI:** 10.1155/2013/934171

**Published:** 2013-08-20

**Authors:** Muhammad Idrees, Ahmad Adnan, Fahim Ashraf Qureshi

**Affiliations:** ^1^Department of Chemistry, GC University, Katchery Road, Lahore 54000, Pakistan; ^2^Office of Research, Innovation and Commercialization, COMSATS Institute of Information Technology, Chak Shahzad, Park Road, Islamabad 45600, Pakistan

## Abstract

Potential of sodium sulfide and sodium sulfite, in the presence of sodium hydroxide was investigated to pretreat the corncob (CC), bagasse (BG), water hyacinth and rice husk (RH) for maximum digestibility. Response Surface Methodology was employed for the optimization of pretreatment factors such as temperature, time and concentration of Na_2_S and Na_2_SO_3_, which had high coefficient of determination (*R* ^2^) along with low probability value (*P*), indicating the reliable predictability of the model. At optimized conditions, Na_2_S and Na_2_SO_3_ remove up to 97% lignin, from WH and RH, along with removal of hemicellulose (up to 93%) during pretreatment providing maximum cellulose, while in BG and CC; 75.0% and 90.0% reduction in lignin and hemicellulose was observed. Saccharification efficiency of RH, WH, BG and CC after treatment with 1.0% Na_2_S at 130°C for 2.3–3.0 h was 79.40, 85.93, 87.70, and 88.43%, respectively. WH treated with Na_2_SO_3_ showed higher hydrolysis yield (86.34%) as compared to Na_2_S while other biomass substrates showed 2.0–3.0% less yield with Na_2_SO_3_. Resulting sugars were evaluated as substrate for lactic acid production, yielding 26.48, 25.36, 31.73, and 30.31 gL^−1^ of lactic acid with 76.0, 76.0, 86.0, and 83.0% conversion yield from CC, BG, WH, and RH hydrolyzate, respectively.

## 1. Introduction 

Conventional substrates such as starch or sucrose are not available in large quantities with affordable prices for the production of industrial chemicals [1]. Lignocellulosic materials are attractive alternatives due to being easily available in large quantities at low price all over the world [2, 3]. They include agricultural residues (corn stover, bagasse, and rice husk), forestry residues (sawdust), portions of municipal solid wastes (waste paper), herbs and shrubs (switchgrass and water hyacinth), woody plants (poplar trees), and various industrial wastes [4]. Agricultural residues such as corncob, bagasse, and rice husk represent large renewable assets for lignocellulosic biomass [5]. According to the Food and Agriculture Organization of the United Nations, worldwide annual production of corn is about 695 ∗ 10^9^ Kg, having 18% corncob [6], available as renewable raw material [7]. Corn is a major food crop after wheat and rice in Pakistan with annual production of 3760 × 1000 MT during year 2010-2011 [8]. Bagasse obtained after juice extraction consists of 40.0%–50.0% cellulose, 25.0% hemicellulose, and 20.0% lignin, which could be converted into fermentable sugars. In Southeast Asia, 216 million ton agricultural biomass comprises of rice [9] which has 20.0% of rice husk [10]. Transformation of these agricultural byproducts into fermentable sugars and finally into lactic acids is desirable. Lactic acid has the best rudiments to become an intermediary product because its functional groups make it prone to further reactions, for example, the production of propionic acid, acetic acid, acrylic acid, alanine acid, or pyruvic acid [11].

The conversion of lignocellulosic materials into fermentable sugars usually includes high temperature (100–200°C) with or without use of chemicals, which leads to the production of compounds such as lignin, acetic acid, furfural, and 5-hydroxymethylfurfural [12, 13] that are toxic to microorganisms. Enzymatic hydrolysis is an interesting way to produce sugars from cellulosic wastes because it requires mild operating conditions such as pH and temperature, without production of byproducts [14, 15]. It requires a pretreatment process due to the recalcitrant nature of lignocellulosic materials, making the carbohydrate more accessible to the hydrolytic enzymes. Various physical (mechanical irradiation, microwave irradiation, and pyrolysis), chemical (acids such as sulfuric acid, phosphoric acid, maleic acid; alkalis such as sodium hydroxide, ammonia, ammonium sulfite; organic solvents such as ethanol/butanol/benzene-water and different swelling agents, oxidation with hydrogen peroxide, ozone, and wet oxidation), physiochemical (steam explosion, carbon dioxide explosion, and ammonia fiber explosion), and biological pretreatment techniques have been proposed, but all of them differ due to a change in the nature of the biomass [16–18]. There are a need to optimize the pretreatment method by choosing a suitable chemical which will provide maximum enzymatic hydrolysis [19]. Traditional sulfide and sulfite pulping processes have been in industry practice with 100 years of experience, providing a lot of knowledge and process expertise [20]. Sulfite treatment decreases the crystallinity of the xylan and the cellulose [21–24] and increases the hydrophilicity of the lignin though sulfonation [20]. Sodium hydroxide treatment reduces the lignin content 60%–90% at varying concentrations (1%–8%) along with decreasing the crystallinity of the cellulose by disrupting the structural linkages and increasing the surface area for enzyme penetration [25–28]. For maximum digestibility of the agricultural residues into fermentable sugars, combined effect of NaOH and Na_2_S or Na_2_SO_3_ could be a potential solution for the removal of lignin, depolymerization of xylan without degrading the cellulose.

 This study focused on alkali-catalyzed sulfide and sulfite pretreatments in order to optimize the pretreatment conditions by using the response surface methodology. The objectives of this study were the following: (1) finds the suitable catalyst for pretreatment of biomass, (2) statistical determination of the optimized pretreatment conditions at the lowest catalyst concentrations (%), pretreatment time, and temperature, and (3) lactic acid production from resulting sugar.

## 2. Material and Method

### 2.1. Chemicals and Biomass

All chemicals were of analytical grade and used without further purification. ACCELLERASE 1500 and OPTIMASH BG were obtained from Genencor International Inc. Corncob, bagasse, water hyacinth, and rice husk were collected from different areas in Pakistan during 2011. These biomass materials were chopped into small pieces (1-2 cm) and dried in a hot-air oven at 105°C for 6 h. Finally, the cleaned and dried biomass materials were ground into powdered form separately. The dried powder material was reserved at room temperature for further work.

### 2.2. Optimization of Pretreatment Process

The catalyst {NaOH (0.5% in water) catalyzed Na_2_S or Na_2_SO_3_} concentration (*X*_1_), time (*X*_2_) and temperature (*X*_3_) used for pretreatment were optimized by using central composite design experiment to enhance the enzymatic hydrolysis yield. The design matrix with eighteen experimental runs in two blocks with four replicates of the midpoint was used. Twenty-five grams of each substrate (CC, BG, RH, and WH) powder was mixed with predefined catalyst solution at 1 : 10 (w/v) ratio in 500 mL flask separately. The flasks were autoclaved (CL-40L) (ALP Co., Ltd., Tokyo Japan) at different temperatures (90–130°C at pressure 0.13–0.2 MPa) for different time intervals as defined through RSM. The solutions in flasks were cooled and neutralized to pH 5-6 using 5.0% sulfuric acid solution, and they then filtered with Whatman filter paper 1. The residues were washed with distilled water for several times to remove the excess alkali or acid and dissolved byproducts that might inhibit the enzymes in subsequent hydrolysis. The residue was dried at 105°C for 20 min. and weighed. Coded values of independent variable along with their minimum and maximum values are shown in Table 1. The model, used to enhance the response by optimizing the pretreatment factors, was a second-order polynomial as follows:

(1) Y=β0+∑i=1nβiXi+∑i=1nβiiXi2+∑i=1n∑j=1nβijXiXj,

where *Y* is the hydrolysis yield and *i*, *j* are the linear and quadratic coefficients, respectively; *β*_0_ is the regression coefficient, and *X*_1_–*X*_3_ are the coded factors under study. Regression analysis and estimation of the coefficients were performed using Design Expert Software 8.1.07. 

### 2.3. Enzymatic Hydrolysis of Pretreated Materials and Lactic Acid Fermentation

Accellerase 1500 and Optimash BG were used for hydrolysis of the pretreated substrate as describe by Idrees et al. [29]. *Lactobacillus acidophilus*, a homofermentative, lactic acid producing bacteria [30] was used for the production of lactic acid from enzymatic hydrolyzate. Inoculum was prepared by transferring cells into 100 mL flask containing 50 mL of culture medium containing 10.0 g/L yeast extract, 2.0 g/L (NH_4_)_2_HPO_4_, 0.10 g/L MnSO_4_ and 30.0 g/L glucose, and it was subsequently incubated at 37°C for 12.0 h [31]. After two consecutive transfers to fresh medium, this was used to inoculate the fermentation medium. Cellulosic hydrolyzates, obtained from enzymatic hydrolysis of each pretreated sample, were used for fermentation. The inoculum to solution ratio of 1 : 20 was used for fermentation purposes. Samples, for glucose and lactic acid analysis, were taken at specific intervals during 72.0 h fermentation process. 

### 2.4. Analysis of Biomass Components, Reducing Sugars and Lactic Acid

#### 2.4.1. Biomass Components

The amount of extractives was determined with acetone using the Soxhlet apparatus. Two grams of dried biomass was used with 120 mL acetone for extraction at 90°C for two hours. After extractions solvents were removed, and the remaining residues dried at 105°C. From weight differences before and after the extraction, calculate the amount of the extractives [32, 33]. The quantification of hemicellulose was done by heating 2.0 grams extractive free dried biomass samples for two hours at 80°C in 20.0 mL of 0.5 mol solution of sodium hydroxide. After that, they washed with deionized water for neutral pH (pH = 7) and dried to a constant weight. The differences in biomass weight are the amounts of the hemicellulose [32, 33]. To determine the amount of the lignin, 1.0-gram extractive-free biomass sample were added in 30.0 mL of 98% sulfuric acid and kept for twenty-four hour at ambient temperature, then boiled at 100°C for one hour. The mixtures were filtered, and the residues were washed with deionized water until complete removal of the sulfate ion. They were then dried to a constant weight which is the weight of the lignin [32, 33]. The cellulose contents were calculated from differences in total weights and the amounts of the hemicellulose, lignin and extractives by assuming that these are the only components of the entire biomass [32–34].

#### 2.4.2. Reducing Sugars

Reducing sugars were determined by using Ghose (1987) [35] method; 3, 5-dinitrosalicylic acid (DNS) was used as a coloring reagent, and the absorbance for each sample was recorded at *λ* = 546 nm with double-beam spectrophotometer (Cecil 7200). Identification of monosaccharide contents was determined with the help of thin-layer chromatography (TLC) in pretreated, enzymatic hydrolyzate and fermentation media [29]. Sugar yield was calculated on pretreated solid biomass used for enzymatic hydrolysis by using the following equation [36]:

(2) sugar  yield  (%) =100(  sugar  produced  during  hydrolysisgram  of  biomass  feedstock).

#### 2.4.3. Lactic Acid

Lactic acid was quantified with the help of HPLC (LC-20AT, Shimadzu) method used by Bai et al., 2000 [37]. Reverse-phase SMA-C18 column with size 4.6 × 250 mm of SMT, coupled with a UV variable wavelength detector (SPD-M20A, Shimadzu) at 210 nm, was employed. The elution was carried out with the help of 0.01 M phosphoric acid having pH 2.5 using an isocratic elution with a flow rate of 1 ml/min at room temperature. The lactic acid has retention time (*t*_*R*_) at 2.4 min.

## 3. Results and Discussion

**Figure 1:**
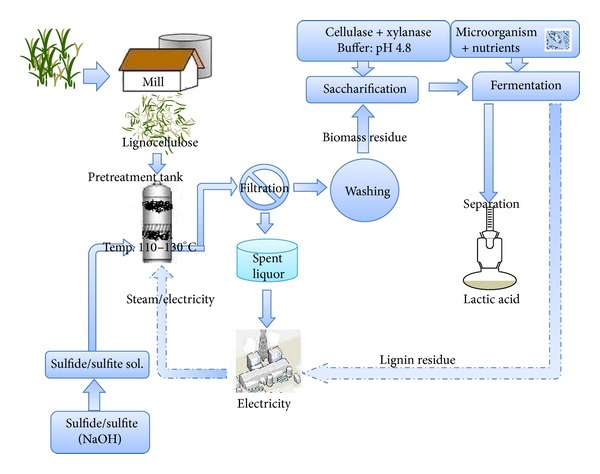
Flow diagram of the process.

Feedstock with low lignin (<20%) content was desirable for maximum hydrolysis yield. Corncob, bagasse, rice husk, and water hyacinth were used as lignocellulosic materials for production of fermentable sugars. Composition of these biomass sources is described in Table 2. Amount of cellulose in bagasse sample was higher (41.0% dry wt. also reported by Sun et al., 2004) [38], as compared with that of water hyacinth which was only 20.39% [39, 40]. Water hyacinth has 42.29% hemicellulose which was less as compared with the 48.7% one reported by Magdum et al., 2012 [41] but it equal to the corncob [42] and greater than bagasse and rice husk. Cellulose content of corncob was 38.98% which was close to the results of Satimanont et al., 2012 [43] while rice husk shows 33.4% cellulose which was 2% less to that reported by Ugheoke and Mamat 2012 [44]. Water hyacinth (WH) and rice husk (RH) have higher amount of extractives (~20%) as compared with corncob and bagasse (~7%). Total amount of polysaccharide were in these substrates are 82.40 > 74.47 > 62.66 > 57.78% corresponding to corncob, bagasse, WH, and RH, respectively. WH has less lignin content (4.23%) as compared to other substrates which range from 13% to 20% on dry-weight basis. Maximum hydrolysis of the biomass was achieved by two-step process, that is, pretreatment and enzymatic hydrolysis. The conditions chosen for pretreatment method will affect various substrates characteristics, which in turn, govern the susceptibility of the substrates to hydrolysis and the subsequent fermentation of the released sugars. So, a careful study was done to pretreat the lignocellulosic materials for maximum digestibility of the cellulose to fermentable sugars and finally fermentation into lactic acid and bioethanol. Schematic diagram of the process was shown in Figure 1.

### 3.1. Effect of Sodium Sulfide and Sulfite on the Biomass Weight Loss (Lignin and Hemicellulose Removal)

**Figure 2:**
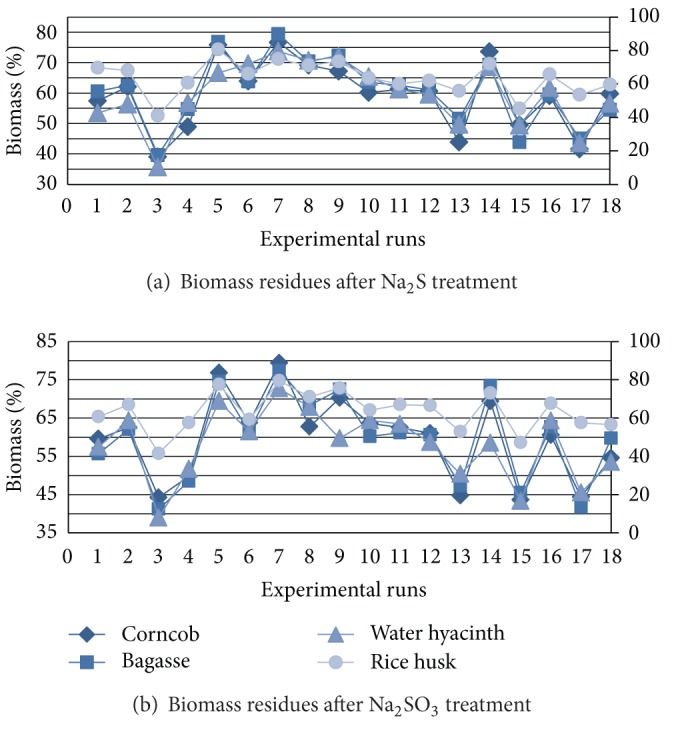
Biomass residues during different experimental runs: (a) when biomass was treated with Na_2_S; (b) when biomass was treated with Na_2_SO_3_.

**Figure 3:**
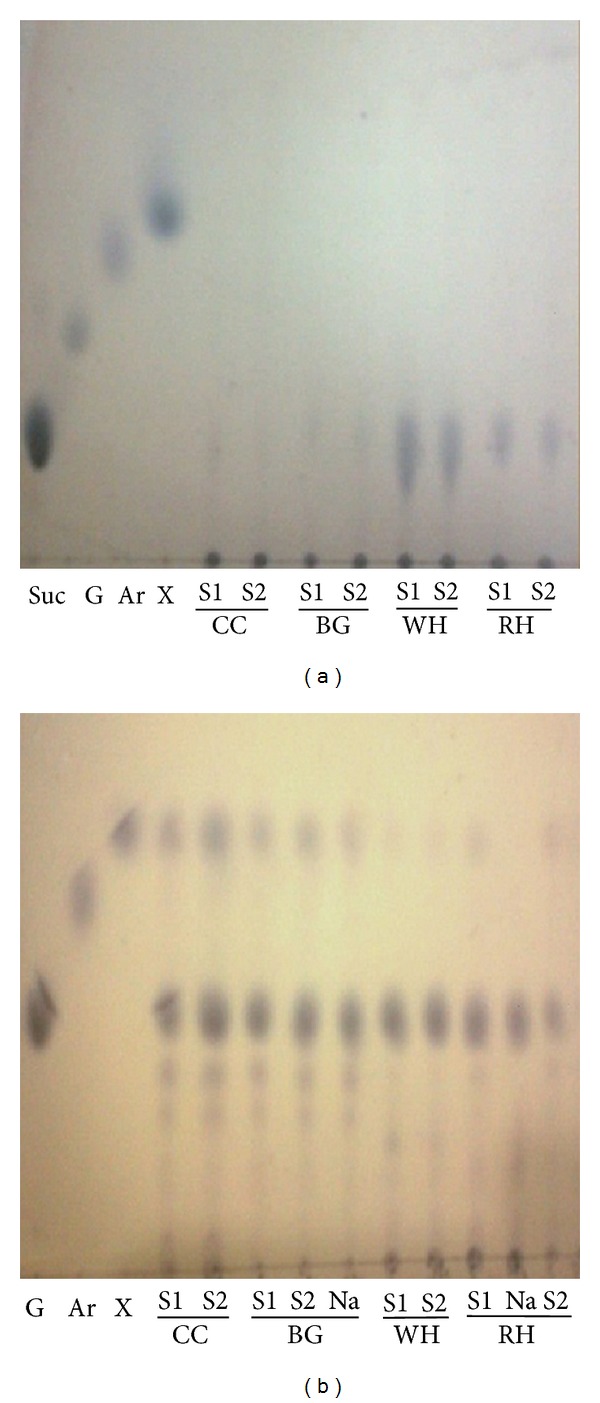
TLC images showing monomeric sugars in hydrolyzates (a) pretreated at 130°C for 3 h; (b) after enzymatic hydrolysis (Suc: sucrose, G: glucose, Ar: arabinose, X: xylose, S1: sodium sulfide, S2: sodium sulfite, and Na: NaOH).

**Figure 4:**
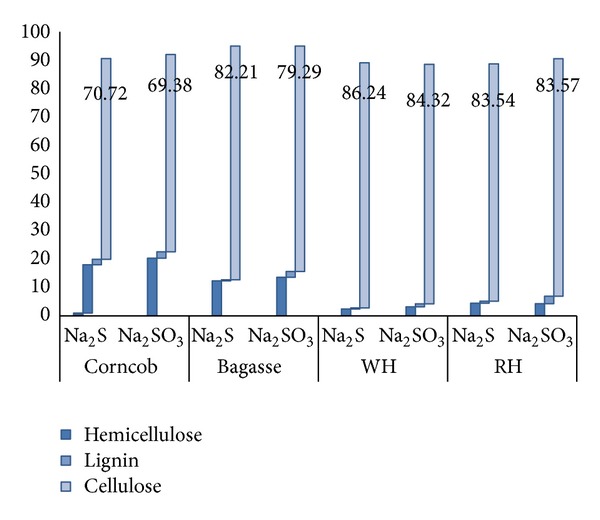
Composition of biomass after optimized pretreatment conditions.

The amount of cellulosic residue after pretreatment was found to be different for different catalysts at varying conditions (Figure 2). The biomass weight loss accounted for the removal of lignin and extractives or hydrolysis of the hemicellulose. The order of decreasing the biomass weight during pretreatment step was WH > BG > CC > RH. More weight loss observed with Na_2_S as compared with Na_2_SO_3_. WH shows 26.32%–65.73% weight loss, while corncob and bagasse show 20.21%–60.38%. Less lignin content of water hyacinth and more hemicellulose hydrolysis, results in more weight loss during pretreatment. Minute quantity of sucrose was present in the pretreated hydrolyzates of BG and RH (Figure 3(a)). Amount of hemicellulose present in pretreated corncob was 15.61% followed by that of bagasse which was 10.46% (dry wt.), while WH and RH show only up to 2% hemicellulose after pretreatment. Water hyacinth and rice husk shows more hydrolysis of hemicellulose (>95%) as compared to corncob and bagasse (>75%). Ndazi et al. 2007, revealed that 4.0%–8.0% NaOH, removed 96.0% lignin and 74.0% hemicellulose during pretreatment of the RH, which increases the amount of cellulose (~80%) in the remaining biomass residue [45]. More hydrolysis of the hemicellulose in pretreated water hyacinth and rice husk was also confirmed by minute quantity of xylose in the enzymatic hydrolyzates (Figure 3(b)). Reduction in lignin content reached up to 97.54% in pretreatment step which was greater than NaOH (1.0%) treatment (85.0%) [16, 26]. In BG and WH, lignin was almost completely removed when pretreated with Na_2_S. Na_2_S has dominant effect on the removal of the lignin as compared with Na_2_SO_3_. After removal of lignin and hemicellulose at optimized conditions, amount of cellulose increased in the residual biomass in different proportions (Figure 4). Na_2_S-treated water hyacinth biomass shows 86.63% cellulose followed by 84.51% from Na_2_SO_3_ treatment and rice husk which provides 83.79% from both treatments. BG shows 79.45% and 82.56% cellulose after Na_2_SO_3_ and Na_2_S treatment, respectively, while corncob shows 70.85% cellulose. In all treated biomass substrates, nearly 85.0%–95.0% polysaccharide material was present for enzymatic hydrolysis. 

### 3.2. Effects of Independent Variables on Hydrolysis Yield

**Figure 5:**
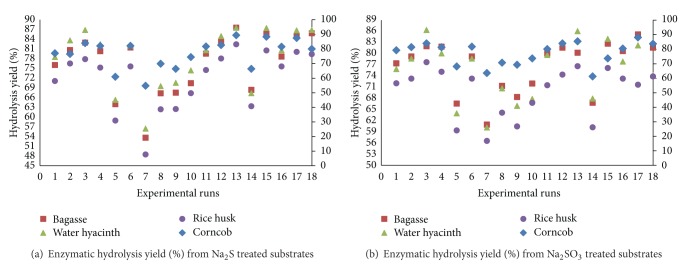
Hydrolysis yield during different experimental runs: (a) when biomass was treated with Na_2_S; (b) when biomass was treated with Na_2_SO_3_.

The enzymatic hydrolysis yield of corncob biomass was greatly influenced by the temperature and time of pretreatment. Hydrolysis yields obtained after different pretreatments with Na_2_S and Na_2_SO_3_ were shown in Figure 5. CC pretreated with 1.0% Na_2_S produces 55.0% and 86.0% hydrolysis yields at 90°C and 130°C, respectively. There is apparent difference between the hydrolysis yields when the concentration of the catalyst during pretreatment was changed from 1.0% to 3.0% at low temperature, 56.62% and 67.92% yields were obtained when Na_2_S (Figure 5(a)) was used during pretreatment at 90°C, respectively. When 3.0% Na_2_S was used at 130°C, the time of pretreatment has no prominent effect on hydrolysis yield, while at 1.0% concentration, the effect of time was dominant, produces 82.71% and 87.69% hydrolysis yield at 130°C with 1.0% and 3.0% Na_2_S catalysts correspondingly. The hydrolysis of bagasse was affected with the temperature of pretreatment and duration of time along with the catalyst concentration as well. At high temperature and low concentration (1.0%) of Na_2_S, hydrolysis yield obtained were 80.57% and 87.41% when duration of time was 1.0 h and 3.0 h, respectively, while at low temperature 53.28% and 63.46% hydrolysis yield was achieved. At high concentrations of Na_2_S there was no much difference in the hydrolysis yield due to duration of time for pretreatment. In case of Na_2_SO_3_ pretreatment, only at lower catalyst concentration, time was affected on the hydrolysis yield at lower temperature but at high temperature difference was not prominent in hydrolysis. At maximum and minimum concentration of catalyst, temperature has a major effect on the hydrolysis yield providing 61.41%–68.36% and 82.51% yield at 90°C and 130°C, respectively. Enzymatic hydrolysis of WH biomass was favored at high temperature and longer time of pretreatment when catalyst (Na_2_S) concentration was low (1.0%), while at high concentration, short time (1.0 h) of pretreatment yielded higher hydrolysis rate (84.81%). At low temperature, 56.35% and 68.71% hydrolysis yield was obtained from 1.0% and 3.0% catalyst concentration, respectively, while at 130°C, 85.0% enzymatic hydrolysis yield was obtained from sodium sulfide (1.0%). Only the effect of temperature was prominent on hydrolysis yield when 3.0% of Na_2_SO_3_ was used during pretreatment step, providing 71.62% and 84.64% hydrolysis yields at 90°C and 130°C, respectively. With 1.0% concentration of Na_2_SO_3_ (Figure 5(b)), the effect of both temperature and time was prominent (79.32 and 86.54% yields). The hydrolysis of the RH was affected with the temperature of pretreatment and duration of time along with the catalyst concentration. High temperature (130°C) and 1.0% concentration of Na_2_S provided 76.43% and 81.53% hydrolysis yields when pretreatment time was 1.0 h and 3.0 h, respectively, while at 90°C, 58.37% yield was achieved. At high concentration of Na_2_S there was no difference in the yield due to time of pretreatment; only temperature improves the hydrolysis yield. In case of Na_2_SO_3_ pretreatment, catalyst concentration along with temperature has a major effect on the hydrolysis yield providing 56.82%–64.57% and 78.17% yield at 90°C and 130°C, respectively. 

### 3.3. Response Surface for the Interaction of Catalyst Conc., Temperature, and Time on Hydrolysis Yield

**Figure 6:**
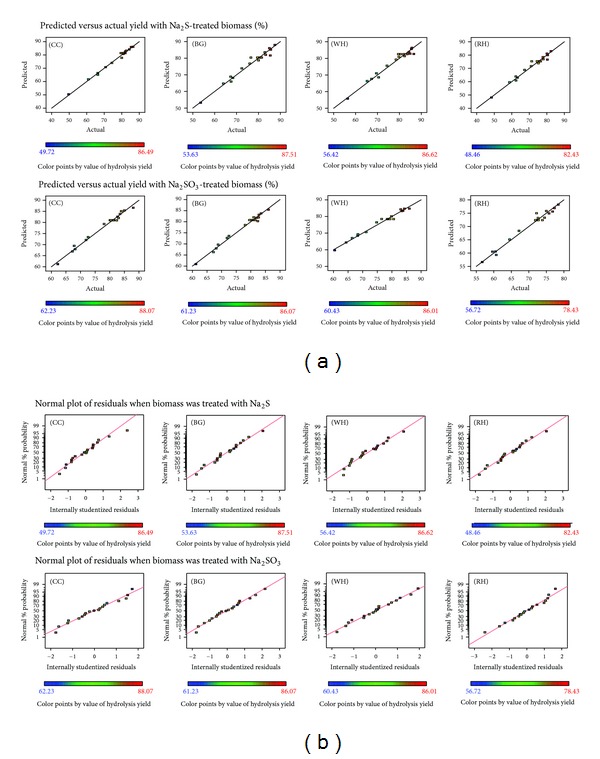
Predicted versus actual yield and normal plots of residuals obtained for different substrates.

The fitness of the model was verified through different diagnostic checks. Residuals were found to follow the normality, and the plots of predicted versus actual yields (Figures 6(a) and 6(b)) also ascertained the overall fitness of the suggested model. Analysis of variance (ANOVA) for the quadratic models described that these models were sufficient to express the actual relationship between the response and significant variables, with a satisfactory coefficient of determination *R* ^2^  {WH: *R* = 0.96^(a)^ and 0.98^(b)^; RH: *R* = 0.96^(a)^ and 0.97^(b)^; BG: *R* = 0.96^(a)^ and 0.98^(b)^; CC: *R* = 0.98^(a)^ and 0.98^(b)^ for Na_2_S^(a)^ and Na_2_SO_3_^(b)^, resp.}. These values which were in reasonable agreement with the adjusted *R* ^2^ indicated 93.0%–98.0% of the variability in the response ensuring a satisfactory adjustment of a quadratic model to the experimental data. Significance of linear terms, that is, catalyst concentration (%), time (h), and temperature (*t*) for pretreatment, was found to be important as their *P* values (prob > *F*) obtained were < 0.05. Also, the *P*-value of the lack of fit for CC: 0.2431^(a)^, 0.0880^(b)^; BG: 0.4355^(a)^, 0.2060^(b)^, WH: 0.2497^(a)^, 0.1991^(b)^; RH: 0.0832^(a)^ and 0.1357^(b)^, respectively, confirmed that the polynomial models fit the processing (Table 3). 

### 3.4. Optimization of Pretreatment Factors for Maximum Hydrolysis Yield

In order to optimize the reaction conditions for the production of high enzymatic hydrolysis yield, RMS was used for experimental purpose. After performing the enzymatic hydrolysis of pretreated materials obtained from the reaction conditions predefined by the RSM and statistical analysis of the data obtained, numerical and graphical optimization was performed. A variety of pretreatment conditions, with desirability 1.0 were selected for maximum hydrolysis yield at lowest catalyst level (1.0% catalyst). When CC was used as lignocellulosic substrate for production of fermentation sugars by using Na_2_S^(a)^ as a catalyst, the optimum factors were obtained as follows: 1.0% Na_2_S^(a)^ at reaction temperature of 130°C for 2.34 h and with Na_2_SO_3_^(b)^ concentration, 1.0% at temperature 130°C for 2.30 h. Similarly of maximum hydrolysis yield was achieved from pretreated bagasse, when it treated with Na_2_S^(a)^ (1.0%) at temperature 130°C for 3.0 h and Na_2_SO_3_^(b)^: concentration 1.0% at temperature 130°C for 2.0 h. Correspondingly, maximum hydrolysis yield was achieved from pretreated WH, when it treated with Na_2_S^(a)^ (1%) at temperature 130°C for 3.0 h and Na_2_SO_3_^(b)^: concentration 1.0% at temperature 130°C for 2.39 h. When rice husk was used as biomass source for production of reducing sugars, the maximum yield was obtained using 1.0% Na_2_S^(a)^ at temperature of 128°C for 2.95 h and 1.0% concentration of Na_2_SO_3_^(b)^ at pretreatment temperature of 125°C for 2.78 h. When we use catalyst concentration at maximum level (3.0%), keeping the temperature and time in range, 88–93% hydrolysis yields were obtained. Keeping in view the processing safety, cost effectiveness and inhibition of by products on enzyme action, we have used the catalyst at lowest level (1.0%), which decreases the yield from 93.0% to 88.0%. To validate optimum yield, experiments with specified conditions were performed. The optimized conditions for corncob, bagasse, water hyacinth, and rice husk were mentioned in Table 4. Resultant hydrolysis yields obtained from optimum conditions are CC: 88.42^(a)^ and 86.32%^(b)^, BG: 87.91^(a)^ and 84.06%^(b)^, WH: 85.93^(a)^ and 86.34%^(b)^, RH: 79.40^(a)^ and 77.17%^(b)^ showed that models were predictive and useful for the optimization of the pretreatment conditions. Previously, Jele et al., 2010 [16] obtained 74.0% hydrolysis yield from NaOH-treated switchgrass, and Zhu et al., 2009 [20] obtained 90.0% cellulose conversion yield by using (9.0%) sodium bisulfite along with sulphuric acid for pretreatment of wood chips at 180°C, which has higher severity conditions. When pretreatment was done with Na_2_S, higher hydrolysis yield was obtained due to more lignin removal, depolymerization of the hemicellulose, and decrease in crystallinity of the cellulose as compared to Na_2_SO_3_ which removed slight less lignin during pretreatment. Rice husk yielded less amount of reducing sugars as compared with other biomass sources (Table 4). If we use these pretreatment conditions at bulk scale, the benefit of the process increases as the electricity charges during pretreatment decreases (Zhu et al., 2009) [20] due to lignin and other byproducts which could be used for energy production as described in the flow diagram (Figure 1).

### 3.5. Lactic Acid Production from Enzymatic Hydrolyzate

**Figure 7:**
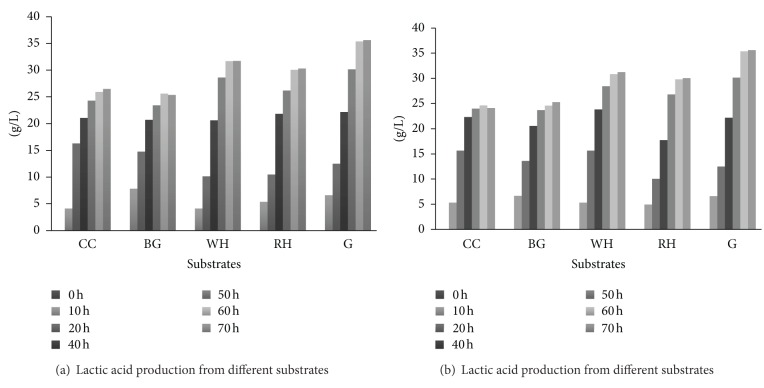
Production of lactic acid form enzymatic Hydrolyzate of different feedstocks; (a): Na_2_S treated substrates, (b): Na_2_SO_3_ treated substrates.

The enzymatic hydrolyzate obtained from the most effective pretreatment condition, containing glucose and xylose was used as a fermenting medium for lactic acid production. The reducing sugars concentration was adjusted at 40.0 gL^−1^ by diluting and concentrating the hydrolyzate. Pure glucose yielded 35.6 gL^−1^ of lactic acid showing 96.0% conversion yield. The enzymatic hydrolyzates of sulfite- and sulfide-treated corncob and bagasse yielded 26.47 and 25.36 gL^−1^ of lactic acid, respectively, while water hyacinth and rice husk yielded 31.71 and 30.31 gL^−1^ of lactic acid (Figure 7). Ali et al., 2009 [46], obtained 25.62 gL^−1^ lactic acid from corncob hydrolyzate through *L. delbrueckii, *while Shen and Xia 2006 [47] obtained 34.40 gL^−1^ of lactic acid in 54.0 h from corncob residue having concentration of 60.0 gL^−1^ with productivity of 0.669 gL^−1^·h^−1^. This difference in the conversion yield was due to the presence of xylose present in the enzymatic hydrolyzate of corncob and bagasse which was not fermented by *L. acidophilus*. These results showed that cellulosic hydrolyzate could be used effectively for the production of lactic acid. Productivity of lactic acid from pure glucose was 0.59 gL^−1^·h^−1^, while enzymatic hydrolyzate of water hyacinth and rice husk showed 0.52 and 0.50 gL^−1^·h^−1^ which were greater than corncob (0.44 gL^−1^·h^−1^) and bagasse (0.42 gL^−1^·h^−1^) hydrolyzate due to the absence of xylose (Table 5). Wee et al., 2006 [31], reported lactic acid productivity 2.2–1.3 gL^−1^·h^−1^ by using the *Lactobacillus* sp. RKY2 on glucose. DNS analysis showed that enzymatic hydrolyzate of water hyacinth and rice husk after lactic acid fermentation showed 2.0–3.0 gL^−1^ of reducing sugars while corncob and bagasse showed 7.64–9.67 gL^−1^ of sugars (xylose) which were the cause of less productivity of lactic acid as compared with the productivity of pure glucose. 

## 4. Conclusions

Corncob, bagasse, water hyacinth, and rice husk have less lignin content and more cellulosic content. These could be potential sources for fermentable sugars which can be achieved through pretreatment and enzymatic hydrolysis. Pretreatment was done with Na_2_S or Na_2_SO_3_ in the presence of NaOH for the removal of lignin and the decreasing of the crystallinity of the substrate for maximum enzyme action. The response surface methodology was employed successfully for the optimization of the pretreatment factors such as catalyst concentration, temperature and time. Keeping in mind the minimum catalyst concentration, optimized level of different factors were: Na_2_S concentration 1.0%, temperature 130°C, time 2.30–3.00 h and Na_2_SO_3_ concentration 1.0%, temperature 130°C and time 2.00–2.78 h. At these conditions, the removal of lignin reached up to 97%. The saccharification efficiency of Na_2_S-treated corncob, bagasse, water hyacinth and rice husk was found to be 88.48, 87.91, 85.93 and 79.40% and the following Na_2_SO_3_; 86.32%, 84.06%, 86.34%, and 77.17%, respectively. Pretreatment with Na_2_S in the presence of NaOH showed higher sugar yield as compared with Na_2_SO_3_ except in WH case. The resulting sugars produced 26.47, 25.36, 31.71, and 30.31 gL^−1^ of lactic acid from corncob, bagasse, water hyacinth and rice husk, respectively, with productivity 0.42–0.52 gL^−1^·h^−1^. This study shows that combined NaOH and Na_2_S/Na_2_SO_3_ pretreatment has outstanding delignification capacity for saccharification of the cellulosic material at minimum concentration, and these biomass sources were considered a potential feedstocks for lactic acid production. 

## Figures and Tables

**Table 1:** Values of independent variables with coded levels during pretreatment steps.

Factor	Name	Units	Type	Minimum	Maximum	Coded	
*A*	Catalyst conc.	%	Numeric	1.00	3.00	−1.000 = 1.00	1.000 = 3.00
*B*	Time	h	Numeric	1.00	3.00	−1.000 = 1.00	1.000 = 3.00
*C*	Temp.	°C	Numeric	90.00	130.00	−1.000 = 90.00	1.000 = 130.00

**Table 2:** Composition of biomass before pretreatment.

Components	Corncob (CC)	Bagasse (BG)	Water hyacinth (WH)	Rice husk (RH)
Extractives (wt% dry)	6.98 ± 0.57	7.65 ± 0.61	17.59 ± 0.82	20.88 ± 0.57
Hemicellulose (wt% dry)	43.42 ± 1.45	33.18 ± 1.03	42.29 ± 1.38	24.20 ± 1.05
Lignin (wt% dry)	13.56 ± 0.74	18.30 ± 0.78	4.23 ± 0.38	20.23 ± 0.74
Cellulose (wt% dry)	38.98 ± 1.05	41.29 ± 1.41	20.37 ± 0.94	33.58 ± 0.94

Duplicate results.

**Table 3:** Analysis of variance (ANOVA) of fitted model.

	Model	Source	Sum of squares	df	Mean square	*F* value	*P* value
							Prob > *F*
Corncob	Na_2_S^(a)^ NaOH (0.5%)	Model	1461.27	9	162.36	72.6	<0.0001 significant
		Residual	17.95	8	2.24		
		Lack of fit	14.45	5	2.89	2.48	0.2431 not significant
		Pure error	3.50	3	1.17		
		Cor total	1479.22	17			
	Na_2_SO_3_^(b)^ NaOH (0.5%)	Model	958.6	9	106.51	67.89	<0.0001 significant
		Residual	12.55	8	1.57		
		Lack of fit	11.39	5	2.28	5.87	0.0880 not significant
		Pure error	1.16	3	0.39		
		Cor total	971.15	17			

Bagasse	Na_2_S^(a)^ NaOH (0.5%)	Model	1392.09	9	154.68	27.98	<0.0001 significant
		Residual	44.22	8	5.53		
		Lack of fit	30.42	5	6.08	1.32	0.4355 not significant
		Pure error	13.80	3	4.60		
		Cor total	1436.32	17			
	Na_2_SO_3_^(b)^ NaOH (0.5%)	Model	834.55	9	92.73	56.90	<0.0001 significant
		Residual	13.04	8	1.63		
		Lack of fit	10.79	5	2.16	2.89	0.2060 not significant
		Pure error	2.24	3	0.75		
		Cor total	847.59	17			

Water hyacinth	Na_2_S^(a)^ NaOH (0.5%)	Model	1189.95	9	132.22	22.37	<0.0001 significant
		Residual	47.29	8	5.91		
		Lack of fit	37.89	5	7.58	2.42	0.2491 not significant
		Pure error	9.40	3	3.13		
		Cor total	1237.25	17			
	Na_2_SO_3_^(b)^ NaOH (0.5%)	Model	990.81	9	110.09	49.57	<0.0001 significant
		Residual	17.77	8	2.22		
		Lack of fit	14.78	5	2.96	2.97	0.1997 not significant
		Pure error	2.99	3	1.0		
		Cor total	1008.58	17			

Rice husk	Na_2_S^(a)^ NaOH (0.5%)	Model	1399.28	9	155.48	27.82	<0.0001 significant
		Residual	44.70	8	5.59		
		Lack of fit	30.15	5	6.03	1.24	0.0832 not significant
		Pure error	14.55	3	4.85		
		Cor total	1443.98	17			
	Na_2_SO_3_^(b)^ NaOH (0.5%)	Model	770.44	9	85.86	34.61	<0.0001 significant
		Residual	19.79	8	2.47		
		Lack of fit	17.29	5	3.36	4.15	0.1357 not significant
		Pure error	2.50	3	0.83		
		Cor total	790.23	17			

**Table 4:** Optimized hydrolysis yield with independent variables used during pretreatment.

Substrate	Catalyst	Catalyst (%)	Time (h)	Temperature (°C)	Hydrolysis yield (%) (pretreatment)	Hydrolysis yield (%) (enzymatic)
Corncob	Na_2_S	1.0	2.34	130.0	n.d.	88.43
	Na_2_SO_3_	1.0	2.30	130.0	n.d.	86.32
Bagasse	Na_2_S	1.0	3.00	130.0	n.d.	87.91
	Na_2_SO_3_	1.0	2.00	130.0	n.d.	84.06
Water hyacinth	Na_2_S	1.0	3.00	130.0	n.d.	85.93
	Na_2_SO_3_	1.0	2.39	130.0	n.d.	86.34
Rice husk	Na_2_S	1.0	2.95	128.0	n.d.	79.40
	Na_2_SO_3_	1.0	2.78	125.8	n.d.	77.17

n.d.: not determined due to dark-color liquor and absence of monomer sugars in TLC images.

**Table 5:** Kinetic parameters of lactic acid fermentations during batch culturing of *Lactobacillus acidophilus*.

Substrate	Reagent for pretreatment	*γ* (initial sugar conc.)	*γ* (lactic acid)	*η* (lactic acid)	Productivity
		gL^−1^	gL^−1^	gg^−1^	gL^−1^·h^−1^
Corncob	Na_2_S	40.0	26.48 ± 0.32	0.76	0.44
	Na_2_SO_3_	40.0	24.11 ± 0.94	0.75	0.40
Bagasse	Na_2_S	40.0	25.27 ± 0.46	0.76	0.42
	Na_2_SO_3_	40.0	25.36 ± 0.52	0.76	0.42
Water hyacinth	Na_2_S	40.0	31.73 ± 0.82	0.86	0.52
	Na_2_SO_3_	40.0	31.21 ± 0.61	0.86	0.52
Rice husk	Na_2_S	40.0	30.31 ± 0.69	0.83	0.50
	Na_2_SO_3_	40.0	30.03 ± 0.47	0.83	0.50
Glucose	No treatment	40.0	35.61 ± 0.86	0.96	0.59

*η* (lactic acid) = lactic acid produced/sugar consumed (g/g).
